# 

**Published:** 1881-10

**Authors:** 


| | PAGE. |
|---|---|
| ORIGINAL COMMUNICATIONS: | |
| Otorrhœa. By F. W. Abbott, M. D., . . | 97 |
| CLINICAL REPORTS: | |
| Elbow Fractures, etc. Reported by Bernard Bartow, M. D., . , . . . | 107 |
| Surgical Cases Treated at Buffalo General Hospital.—Service of Dr. C. C. F. Gay. Reported by C. C. Fredericks, M. D., House Surgeon, . . . . . . | 111 |
| SELECTIONS: | |
| Introductory Remarks. Delivered upon the Opening of the Section on Pathology. By Samuel Wilkes, M. D., F. R. S., . | 114 |
| The Diagnosis and Treatment of Whooping Cough. By Robert Lee, M. D., . . | 120 |
| An Action for Malpraxis in Belgium, . . | 129 |
| Croup Treated by Passing Catheter into the Trachea by the Mouth. By J. Wilson Paton, M. D., . . . . . . | 130 |
| Cremation in Denmark, . . . . . | 131 |
| Effects of Excision of the Syphilitic Chancre, | 132 |
| Education of the People by Physicians, . | 132 |
| CORRESPONDENCE, . . . . | 133 |
| EDITORIAL: | |
| Death of Prof. James P. White, . . . | 137 |
| Correspondence and Clinical Reports, . | 137 |
| The President's Physicians, . . . | 138 |
| Hygienic, . . . . . . . . . | 139 |
| REVIEWS, . . . . . . . | 139 |

